# Meiotic chromosome movements in plants, a puppet show?

**DOI:** 10.3389/fpls.2014.00502

**Published:** 2014-09-29

**Authors:** Javier Varas, Célia Baroux

**Affiliations:** ^1^Departamento de Genética, Facultad de Biología, Universidad Complutense de MadridMadrid, Spain; ^2^Department of Plant Developmental Genetics, Institute of Plant Biology, Zürich-Basel Plant Science Center, University of ZürichZürich, Switzerland

**Keywords:** telomere, SUN, nuclear envelope, bouquet, meiosis

Meiosis is a special type of cell division by which sexually reproducing organisms maintain their chromosome number across generations. This process produces haploid gametes by two successive rounds of cell division preceded by a unique DNA replication event. During the first meiotic prophase, the homologous chromosomes form stable bivalents, a process which implies their recognition, with a subsequent step of intimate alignment (pairing), synapsis (physical association of paired chromosomes by the synaptonemal complex, SC), and recombination (exchange of chromosomal regions).

Usually, homologous chromosomes are physically separated at initiation of prophase I. In many organisms, telomeres initiate a non-random movement at the entrance of meiosis that brings homologs together tethering at the inner surface of the nuclear envelope (NE).

Sad1/UNC-84 (SUN)-domain proteins are inner NE proteins involved in complexes that link cytoskeletal elements with the nucleoskeleton. In this sense, the SUN proteins connect the telomeres in order to generate the chromosome arrangements, acting as the strings for puppet movements. This telomere attachment to the NE and telomere clustering at the transition between leptotene and zygotene (defining a stage called the “bouquet”) are well-known meiotic phenomena (Zickler and Kleckner, 1998). Numerous lines of evidence obtained in several non-plant model species suggest that they are driven by the meiotic cytoskeleton (Figure 1). Also, it has been shown that disruption of the telomere/nuclear envelope attachment during meiosis induces alterations in pairing and synapsis (Kracklauer et al., 2013).

**Figure 1:**
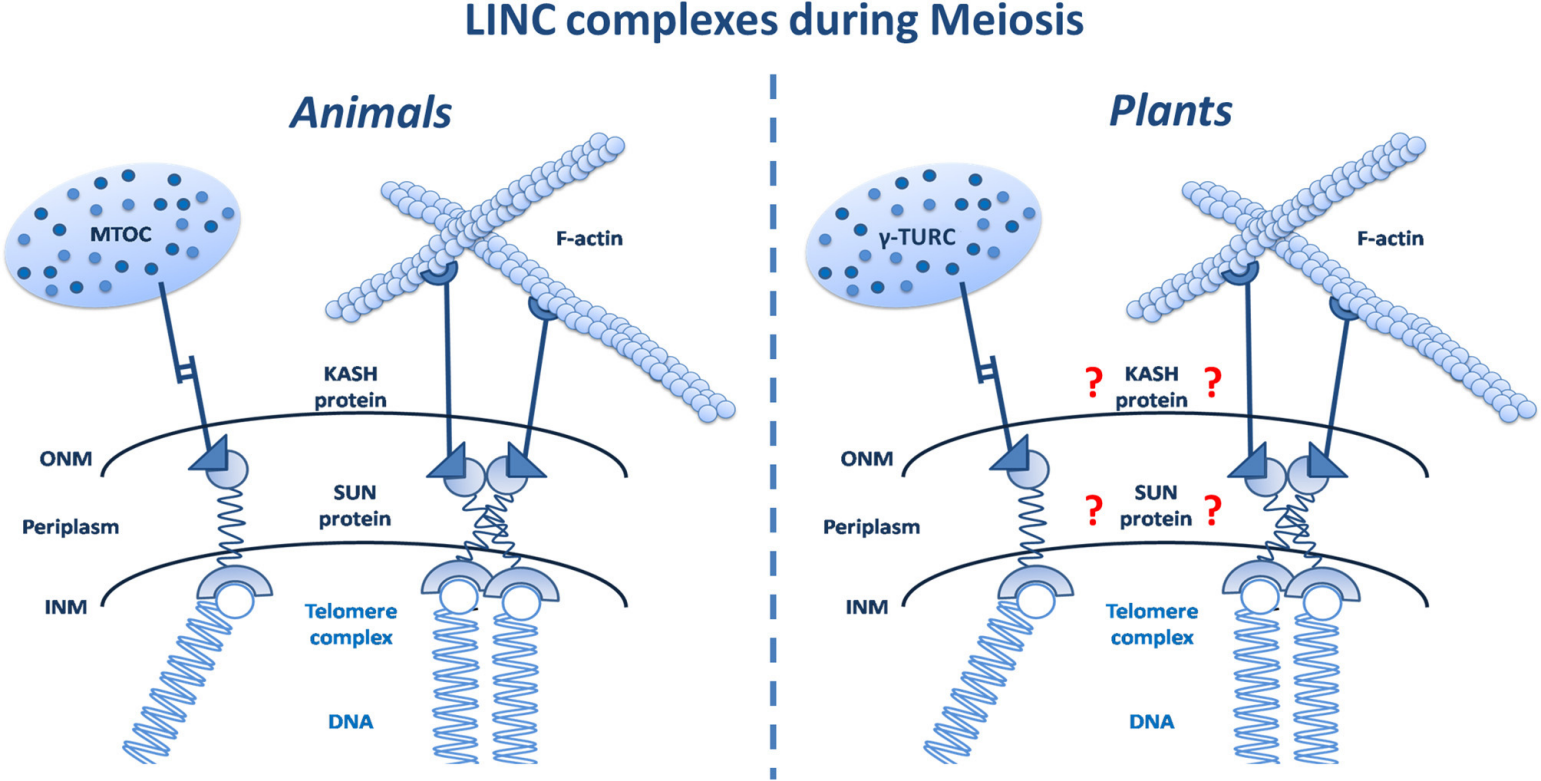
**Working model for the LINC complexes during meiosis**. These, are able to act as molecular bridges in animals (left panel). By contrast, this function remains unknown in plants (right panel). LINC complexes allows the cytoskeleton to regulate the telomere position within the nucleus. (MTOC, microtubule-organizing center; LINC, Linker of Nucleoskeleton and Cytoskeleton; ONM, outer nuclear membrane; INM, inner nuclear membrane; γ-TURC, gamma-tubulin ring complex).

The existence of several SUN domain proteins in plants has been described. Graumann et al. (2010) and Murphy et al. (2010). However, the specific role of these proteins during plant meiosis remained largely enigmatic (Figure 1; Roberts et al., 2013). In this issue of Frontiers in Plant Sci. Murphy et al. (2014) provide new evidences that the SUN proteins could be factors involved in facilitating the chromosome movements during the first meiotic prophase. Observations from the Hank Bass laboratory are focused on cytological analyses of maize SUN domain proteins during meiotic prophase. The authors have developed a new antibody against the two SUN proteins from maize and they demonstrate the existence of a characteristic “SUN belt” around the NE. Furthermore, their data suggest interactions between these SUN proteins and the telomeres when the bouquet formation occurs. The work of Murphy et al. (2014) using three classic maize meiotic mutants, provides further insights into the role of SUN proteins in controlling the chromosome movements in plant meiosis. This study is a first step toward a better understanding of chromosome dynamics during the meiotic program.

## Conflict of interest statement

The authors declare that the research was conducted in the absence of any commercial or financial relationships that could be construed as a potential conflict of interest.
